# A Real-Time Evaluation Algorithm for Noncontact Heart Rate Variability Monitoring

**DOI:** 10.3390/s23156681

**Published:** 2023-07-26

**Authors:** Xiangyu Han, Qian Zhai, Ning Zhang, Xiufeng Zhang, Long He, Min Pan, Bin Zhang, Tao Liu

**Affiliations:** 1 State Key Laboratory of Fluid Power and Mechatronic Systems, School of Mechanical Engineering, Zhejiang University, Hangzhou 310027, China; hanxiangyu@zju.edu.cn (X.H.); 11825031@zju.edu.cn (Q.Z.); 2 National Research Center for Rehabilitation Technical Aids, Beijing 100176, China; zhangning@nrcrta.cn (N.Z.); zhangxiufeng@nrcrta.cn (X.Z.); 3 Zhiyuan Research Institute, Hangzhou 310024, China; helong_zyy@163.com; 4 Department of Mechanical Engineering, University of Bath, Bath BA2 7AY, UK; mp351@bath.ac.uk; 5 Department of Electrical Engineering, University of South Carolina, Columbia, SC 29208, USA; zhangbin@cec.sc.edu

**Keywords:** FMCW radar, heartbeat, HRV, wireless signal, noncontact monitoring, vital sign monitoring, algorithm

## Abstract

Noncontact vital sign monitoring based on radar has attracted great interest in many fields. Heart Rate Variability (HRV), which measures the fluctuation of heartbeat intervals, has been considered as an important indicator for general health evaluation. This paper proposes a new algorithm for HRV monitoring in which frequency-modulated continuous-wave (FMCW) radar is used to separate echo signals from different distances, and the beamforming technique is adopted to improve signal quality. After the phase reflecting the chest wall motion is demodulated, the acceleration is calculated to enhance the heartbeat and suppress the impact of respiration. The time interval of each heartbeat is estimated based on the smoothed acceleration waveform. Finally, a joint optimization algorithm was developed and is used to precisely segment the acceleration signal for analyzing HRV. Experimental results from 10 participants show the potential of the proposed algorithm for obtaining a noncontact HRV estimation with high accuracy. The proposed algorithm can measure the interbeat interval (IBI) with a root mean square error (RMSE) of 14.9 ms and accurately estimate HRV parameters with an RMSE of 3.24 ms for MEAN (the average value of the IBI), 4.91 ms for the standard deviation of normal to normal (SDNN), and 9.10 ms for the root mean square of successive differences (RMSSD). These results demonstrate the effectiveness and feasibility of the proposed method in emotion recognition, sleep monitoring, and heart disease diagnosis.

## 1. Introduction

Heart Rate Variability (HRV) describes the variation in the time interval between successive heartbeats. It is generally considered to be the result of an interaction between the heart and the brain, which is also called neuro-cardiac function. HRV is controlled by the autonomic nervous system (ANS), including the parasympathetic nervous system (PNS) and sympathetic nervous system (SNS). In recent years, research on HRV has attracted lots of interest from the fields of instrumentation [1], signal processing [2], and healthcare [3]. Studies have confirmed that HRV indicators can be applied to sleep monitoring [4] and the diagnosis of some cardiovascular diseases [5]. They can also be used to evaluate people’s mental stress [6] and can even further help home buyers choose residences [7].

### 1.1. Background

Electrocardiography (ECG) and photoplethysmography (PPG), which reflect the change in body surface electrical potentials and blood volume fluctuations in a superficial body location, respectively, are still the mainstream monitoring methods for HRV [8]. However, as contact measurement methods, they both have many limitations in practice. Their lead wires will hinder the user’s physical movement. For electrocardiographs, many electrodes need to be attached to particular locations, which complicates the monitoring process, makes people feel uncomfortable, and puts higher requirements on operators. In addition, they do not apply to certain groups, such as infants, patients with skin burns, and individuals with sleep disorders [9,10,11,12,13].

In the past 50 years, contactless physiological monitoring has developed rapidly [14] and has largely overcome the disadvantages of contact equipment such as electrocardiographs. It can monitor respiration, heartbeat, or other physiological signals without directly contacting the body of the monitored subject and will not make them feel uncomfortable or interfere with their daily routine [15], which allows its use for special groups like infants. Monitoring based on millimeter-wave radar is considered a promising noncontact physiological monitoring method that can penetrate non-metallic obstacles, such as clothes or quilts on the surface of the monitored subject, to capture physiological signals of the human body [16]. It can be used to probe respiration disorders, such as obstructive sleep apnea (OSA) and sudden infant death syndrome (SIDS), as well as used in medical sleep labs and earthquake or fire search-and-rescue scenarios [17,18]. The radiofrequency (RF) signal is more robust to temperature changes or environmental thermal noise compared with infrared thermal imaging methods [12] and is better able to avoid insufficient image resolution, blind areas, or potential privacy problems compared with vision-based monitoring methods [19].

However, measuring the time interval between successive heartbeats and analyzing HRV remain significant challenges due to the weak amplitude of the heartbeat signal and interferences from respiration, trunk movement, and various noises. Most of the existing research in this field focuses on monitoring the respiration rate and heartbeat rate [20], while only a few studies focus on the extraction of HRV characteristics.

### 1.2. Related Works

In 2007, Massagram et al. successfully extracted the time interval between successive heartbeats from echo signals of continuous-wave (CW) radar, proving the feasibility of monitoring HRV with the RF method [21]. Early studies on this subject mainly chose CW radar as RF equipment [16,21]. In some of these studies, the peak corresponding to the heartbeat signal can be clearly distinguished in the demodulated chest movement signal. So, the heartbeat signals can be easily separated by using some filters or using classical signal processing analysis methods, such as autocorrelation, from which the time interval between successive heartbeats can be extracted so that HRV can be measured [22,23,24]. However, CW radar has a weak anti-interference ability. When other subjects appear in the monitoring range, the movement of one individual will affect the measurement and extraction of physiological signals from the other individual, leading to a large measurement error.

FMCW radar provides an important approach to solve this problem. The modulated frequency provides a unique range resolution that CW radar does not have, which enables the separation of the echo signals of objects at specific distances. As a result, FMCW has a strong anti-interference ability and the potential for monitoring multiple subjects simultaneously [25,26]. Effective algorithms for determining the distance between monitored subjects and radar were also developed [27,28,29]. When there are multiple individuals at the same distance from the radar, their vital signals can still be separated and monitored simultaneously with beamforming technologies [30,31,32].

In practice, the amplitude of the heartbeat waveform demodulated from the FMCW radar echo signal is weak: it is orders of magnitude smaller than the amplitude of the respiration waveform and almost buried in a combination of harmonic respiration signals and noise in the frequency spectrum of the phase sequence. Therefore, the design of the heartbeat signal extraction algorithm plays an important role in monitoring.

A bandpass filter is a common method to separate heartbeat signals from demodulated chest wall motion signals because it is simple to design and easy to implement [23,33,34]. However, it is possible to mistake the harmonics of respiration for heartbeat signals because of their similar amplitudes and frequencies. In addition, it is difficult to accurately extract the time interval of each heartbeat because it is difficult for the filter to eliminate the influence of respiratory harmonics. Most previous studies performed HRV analysis based on the average heart rate over a short time (e.g., 3 s) [33,34]. Empirical mode decomposition (EMD) is another commonly used heartbeat signal extraction algorithm [35,36,37] that decomposes the chest wall motion according to the time scale characteristics of the signal itself. However, this method has limitations, such as mode aliasing and end effects. In addition, selecting the components of the heartbeat signal from the intrinsic mode function (IMF) remains a technical challenge.

With the development of signal processing technology, some novel algorithms were proposed to extract heartbeat signals and achieved accurate results. Lv et al. used the envelope mid-line method to remove the respiration signal and proposed a stochastic resonance algorithm to enhance the amplitude of heartbeat signals [30]. The experimental results were highly consistent with those of PPG detection. The average accuracy of the heart rate value in all subjects reached 96.56%, and the relative error of SDNN was less than 6.53%. Xiong et al. [38] proposed a differential enhancement (DE) method, which uses differential operation to significantly enhance the heartbeat components, especially high-order heartbeat harmonics. Combined with the autocorrelation-based periodicity extraction technique, DE can locate the true heartbeat rate (HR). However, it is challenging to accurately extract the duration of each heartbeat because the waveform after the difference operation is complicated, and it is difficult to segment through peak detection. This problem is addressed by the method proposed by Zhao et al. [39] with the assumption that successive human heartbeats should have the same morphology. Hence, the corresponding heartbeat waveforms should have the same overall shapes, while they may stretch or compress due to different beat lengths. On this basis, the method transforms the above segmentation problem into an optimization problem and solves the optimal “template” while seeking the optimal segmentation. In their experimental results, the extracted time intervals of each heartbeat are within milliseconds of the ECG signal. However, the algorithm requires a lot of interpolation operations during the segmentation process, which makes it difficult to monitor the HRV index in real time.

In this paper, an HRV monitoring algorithm based on FMCW radar is proposed to address the issues of existing methods. It is based on a single-chip multiple-input multiple-output (MIMO) radar sensor, zooms in on human reflections, and neglects reflections from other subjects by using the range resolution characteristics of FMCW radar and beamforming techniques. It was found that the change process of the respiration waveform is slow and steady, while a heartbeat involves rapid contraction of the muscles. So, the second-order difference is calculated to enhance the heartbeat and suppress the impact of respiration. An approximated estimate of the duration of each heartbeat can be obtained by calculating the short-time average power of the acceleration signal. Finally, a joint optimization algorithm was developed and is used to adjust the duration of each heartbeat to meet the accuracy requirements of HRV analysis. Compared with the algorithm in [39], the proposed method is ten times to dozens of times faster. Experiments are presented to verify the performance of the proposed algorithm in accurately extracting HRV characteristics and reducing computation. The proposed algorithm shows great potential for real-time evaluations.

This article is organized as follows: Section 1 introduces the background and the related works and discusses their advantages and limitations. Section 2 presents the proposed algorithm. Section 3 describes the experimental validation protocols, and the results are illustrated and discussed in Section 4. Finally, conclusions are drawn in Section 5.

## 2. Algorithms

**Figure 1:**
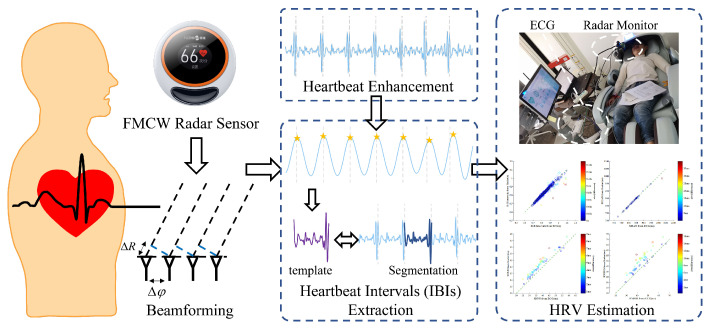
A real-time evaluation algorithm for noncontact Heart Rate Variability monitoring.

Figure 1 shows the main configuration of the proposed method, a real-time HRV index monitoring algorithm based on FMCW radar. The algorithm can effectively extract each heartbeat interval from the echo signal of FMCW radar and accurately estimate the HRV index.

### 2.1. FMCW Radar Signal Preprocessing

For FMCW radar, each transmitting antenna sends one linear frequency-modulated pulse (a chirp) at a time. Chest displacements due to breathing and heartbeat are measured through the phase modulation presented in the output of the mixer, delivering a signal that contains breathing and heartbeat effects. The mathematical expression of the intermediate frequency (IF) signal is denoted by

(1) $$S_{IF}\left(t\right)=A\exp\left[j\left(S\frac{4\pi R}{c}t+\frac{4\pi R}{\lambda }\right)\right].$$

where *A* is the amplitude of the IF signal, *S* is the slope of frequency over time, which is a constant, *c* is the speed of light, and λ is the wavelength of the impulse. *R* is the distance between the chest wall and the antenna.

It is clear from (1) that the frequency *f* and phase φ of $S_{IF}\left(t\right)$ are proportional to the distance between the chest wall and antenna and can be shown as

(2) $$f=S\frac{2R}{c}$$

and

(3) $$\varphi =\frac{4\pi R}{\lambda }.$$

For each chirp, the IF signal is digitized by an analog-to-digital converter (ADC), producing N samples. The components of different frequencies in the output of the mixer can be separated by the fast Fourier transform (FFT). The component reflected by the human chest wall can be selected by (2) if the distance (donated by $R_{0}$) between the chest wall and antenna is known, from the phase of which the movement waveform of the chest wall can be obtained.

**Figure 2:**
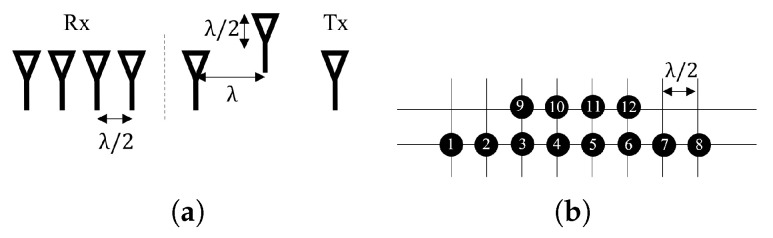
(**a**) The antenna array and (**b**) the corresponding virtual antenna array.

As shown in Figure 2a, the radar equipment in this research has three transmitting antennas and four receiving antennas. The distance between the adjacent transmitting and receiving antennas is λ and λ/2, respectively. The corresponding virtual antenna array is shown in Figure 2b. Based on the virtual antenna, the minimum variance distortionless response (MVDR) beamforming technology was adopted in our study to suppress interference reflected from other objects in the azimuth plane using the antennas numbered 1–8 in Figure 2b. Assuming that the azimuth relative to the antenna array of the monitored subject’s heart is θ, the steering vector α(θ) can be written as  

(4) $$\alpha \left(\theta \right)={\left[1,\exp\left(-j2\pi \frac{d\sin\theta }{\lambda }\right),\exp\left(-j2\pi 2\frac{d\sin\theta }{\lambda }\right)\cdots \exp\left[-j2\pi \left(M-1\right)\frac{d\sin\theta }{\lambda }\right]\right]}^{H}.$$

where d=λ/2. The optimal weight vector can be formulated as

(5) $$\omega =\frac{R_{\mathrm{xx}}^{-1}\alpha \left(\theta \right)}{\alpha^{H}\left(\theta \right)R_{\mathrm{xx}}^{-1}\alpha \left(\theta \right)}.$$

where *M* is the number of virtual antennas, and $R_{\mathrm{xx}}$ is the covariance matrix calculated by the receiving vector of the virtual antenna array.

### 2.2. Heartbeat Signal Enhancement

Although it is convenient to extract the heartbeat signal using a bandpass filter, some details of the heartbeat signal will also be filtered out because the heartbeat waveform is not strictly sinusoidal, and there will be residual respiratory harmonics, which is undesirable for performing HRV analysis.

**Figure 3:**
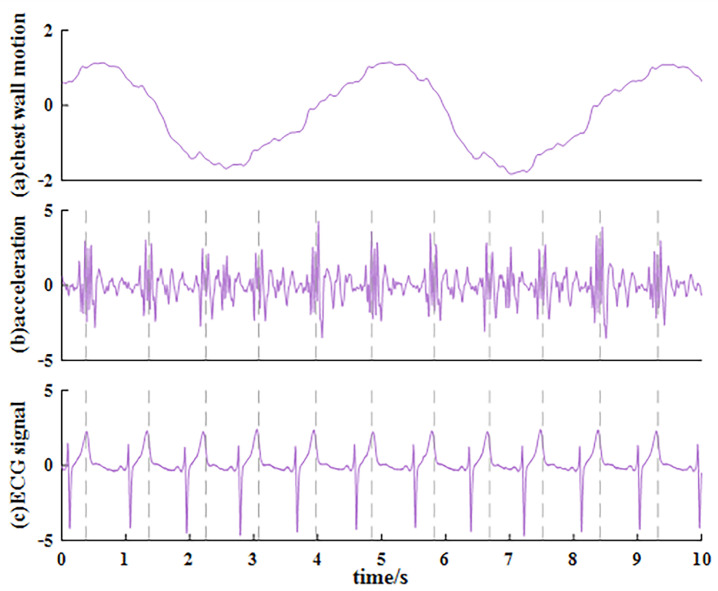
(**a**) An example of chest wall motion signal R(n), (**b**) corresponding acceleration signal, and (**c**) ECG sampled synchronously (all of them are normalized).

We define the chest wall motion signal as R(n). Figure 3a shows an example of R(n), in which distinct breathing movements can be observed, but it is difficult to directly distinguish the vibrations caused by each heartbeat. Considering that the amplitude of vibration caused by the heartbeat in R(n) is small but intense, while the inhale–exhale motion is slow and smooth, the acceleration a(n) (i.e., the second derivative) of R(n) is calculated through (6) to suppress the respiration signal and enhance the heartbeat signal, where $T_{s}$ is the sampling interval. When the sampling frequency is 250 Hz, the corresponding $T_{s}$ is 4 ms.

(6) $$a\left(n\right)=\frac{R(n+1)+R(n-1)-2R(n)}{{\left(T_{s}\right)}^{2}}$$

Figure 3b shows the acceleration of the example shown in Figure 3a. The ECG signal sampled synchronously is shown in Figure 3c. It is clear that the heartbeat signal is enhanced and the breathing motion is dampened.

**Figure 4:**
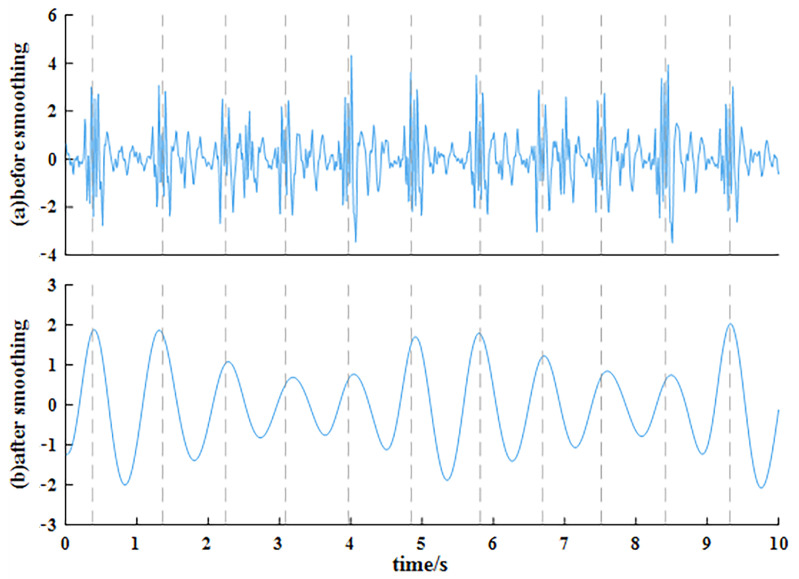
The acceleration signal (**a**) before and (**b**) after smoothing (all of them are normalized, and the dashed line in the figure indicates the location of the spike in the corresponding ECG signal).

Note that while the periodicity of the heartbeat signal can be observed in the acceleration, it is still difficult to pick out each heartbeat cycle by using zero-crossing detection or peak detection because the waveform of the heartbeat in acceleration is not a simple sinusoidal signal, and the morphology of heartbeats in acceleration signals is unknown. For a single heartbeat in acceleration, the middle has a more violent fluctuation degree than the two ends. So, short-time average power P(n) is calculated to describe this feature and smooth the acceleration waveform. P(n) can be calculated as

(7) $$P\left(n\right)=\frac{1}{2L+1}\sum_{j=n-L}^{n+L}{\left|a\left(j\right)\right|}^{2}$$

where *L* is half the length of the time window. If *L* is too small, the smoothing effect is not good, and if *L* is too large, the fluctuation of the waveform after smoothing is not obvious. Based on trail and error, the length of the time window in this paper is 0.4 s, which is about half of the heartbeat cycle. Figure 4 shows the acceleration signal before and after smoothing. The heartbeat signal is sinusoidal after smoothing, so it can be segmented easily. However, Figure 4 shows that many spikes in the smoothed waveform are not completely aligned with the ECG signal, which indicates that some details are lost when the acceleration signal is smoothed. Therefore, the smoothed waveform can only be used to roughly estimate the duration of each heartbeat but does not meet the accuracy requirements for HRV analysis.

### 2.3. Accurate Extraction of Heartbeat Intervals

The variation in the heartbeat time interval is usually a few milliseconds to tens of milliseconds, and the heartbeat interval extracted by the smoothed acceleration waveform does not meet this accuracy requirement. It is necessary to further improve the accuracy of heartbeat intervals for HRV analysis. Notice that although the waveforms of different single heartbeats are very complex, they are very similar in shape. Based on this, we can adjust the location of the cut-off point between two adjacent smoothed heartbeat waveforms. With these cut-off points, the template of a single heartbeat can be identified. This template can be used to further adjust the positions of cut-off points (denoted by S) between any two successive heartbeats. The solving process can be described as the following joint optimization problem:

(8) $$\arg\min_{S,\mu }\sum_{s_{i-1},s_{i}\in S}{∥a\left(s_{i-1}+1:s_{i}\right)-\omega \left(\mu ,s_{i}-s_{i-1}\right)∥}^{2}$$

where $S=\left\{\begin{array}{cccc} s_{1} & s_{2} & s_{3} & \cdots \end{array}\right\}$, $s_{i}$ is the cut-off point in the acceleration signal, $a\left(s_{i-1}+1:s_{i}\right)$ is the sequence from the ($s_{i-1}+1$)-th to the $s_{i}$-th elements of acceleration, and ω(μ,p) indicates that the length of the template μ has been changed to *p* elements by cubic spline interpolation. The goal of the algorithm is to find the optimal segmentation S and template μ that minimize the sum of differences between each subsection and template, as shown in (8).

The optimization of the template and segmentation may require several iterations. In the iterative process, the segmentation and template are updated alternately, while the other one is fixed. During each iteration, the algorithm updates the template given the current segmentation through (9), and then it updates the segmentation with the updated template through (10).

(9) $$\mu^{l}=\arg\min_{\mu }Var\left(S^{l},\mu \right)$$

(10) $$S^{l+1}=\arg\min_{S}Var\left(S,\mu^{l}\right)$$

where

(11) $$Var\left(S,\mu \right)=\sum_{s_{i-1},s_{i}\in S}{∥a\left(s_{i-1}+1:s_{i}\right)-\omega \left(\mu ,s_{i}-s_{i-1}\right)∥}^{2}.$$

The superscripts *l* and l+1 indicate the number of iterations. For (9), it can be proved that the optimal template is given by (12), where *m* is the required length of the template, and *n* is the length of a(n). For (10), considering that the complexity increases exponentially with the length of a(n) when seeking the (l+1)-th optimal segmentation $S^{l+1}$ based on the *l*-th template $\mu^{l}$ in the iteration, dynamic programming is adopted, as shown by (13), which reduces the complexity to a linear increase with the length of a(n).

(12) $$\mu^{l}=\frac{1}{n}\sum_{s_{i-1},s_{i}\in S^{l}}\left(s_{i}-s_{i-1}\right)\omega \left(a\left(s_{i-1}+1:s_{i}\right),m\right)$$

(13) $$D_{s_{i}}=\min\left\{D_{s_{i-1}}+{∥a\left(s_{i-1}+1:s_{i}\right)-\omega \left(\mu ,s_{i}-s_{i-1}\right)∥}^{2}\right\}$$

where $s_{i}$ is a cut-off point, and $s_{i-1}$ is a possible cut-off point, which is a specific range (e.g., 0.5–1.2 s) ahead of $s_{i}$. However, if we select $s_{i}$ and $s_{i-1}$ by using a traversal search, the amount of calculation will still be significant because a lot of interpolation operations are needed. In the proposed algorithm, it is assumed that the cut-off points in the current iteration can only appear next to the corresponding cut-off points that were obtained last time. Therefore, it is not necessary to include any two points within the range of the heartbeat cycle (e.g., 0.5–1.2 s) in the search for optimal segmentation. The real cut-off points can be found by just exploring the combination of points in the neighborhood of the estimated cut-off points from the smoothed a(n) or the last iteration, which can be written as

(14) $$\left\{\begin{array}{c} S^{l+1}=\arg\min_{S}Var\left(S,\mu^{l}\right)\\ s_{i-1}^{l}-B\leq s_{i-1}^{l+1}\leq s_{i-1}^{l}+B,\\ s_{i}^{l}-B\leq s_{i}^{l+1}\leq s_{i}^{l}+B \end{array}\right.$$

where *B* is half the length of the neighborhood. The selection of the neighborhood size needs to take stability and computation into account. Too small a neighborhood size may screen the real cut-off point and lead to difficulties in converging. On the contrary, if the selected neighborhood is too large, the computation will increase when exploring the optimal segmentation, as the complexity will increase with the square of the neighborhood size. In this paper, *B* is selected as 20 ms. The value of *B* is 5 when the sampling frequency is 250 Hz.

### 2.4. Algorithm Summary and Convergence Conditions

**Figure 5:**
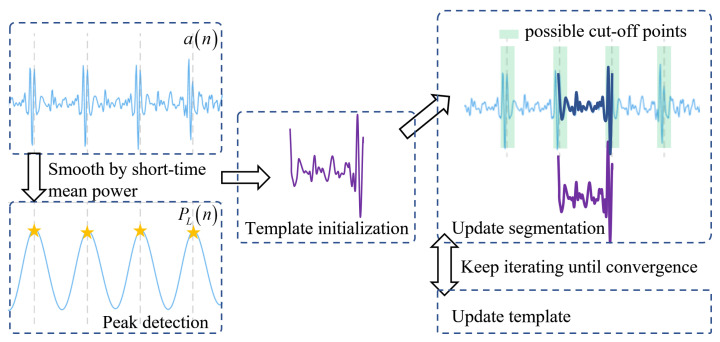
The schematic diagram of the proposed algorithm.

Figure 5 describes the algorithms for strengthening heartbeat signals and extracting heartbeat intervals. The algorithm can also be expressed by Algorithm 1.
**Algorithm 1** Heartbeat Interval Extraction**Require:** The demodulated chest wall motion signal R(n) **Ensure:** The optimal segmentation S
1:$a\left(n\right)\leftarrow \frac{R(n+1)+R(n-1)-2R(n)}{{\left(T_{s}\right)}^{2}}$2:$P\left(n\right)\leftarrow \frac{1}{2L+1}\sum_{j=n-L}^{n+L}{\left|a\left(j\right)\right|}^{2}$3:$S^{0}\leftarrow$ search for peaks in P(n)4:**while** convergence condition is not satisfied **do**5:    $\mu^{l}\leftarrow \frac{1}{n}\sum \left(s_{i}^{l}-s_{i-1}^{l}\right)\omega \left(a\left(s_{i-1}^{l}+1:s_{i}^{l}\right),m\right)$6:    **for** $s_{2}^{l}:s_{end}^{l}$ **do**7:        calculate all possible $D_{s_{i-1}^{l+1}}$8:        where $s_{i-1}^{l}-B\leq s_{i-1}^{l+1}\leq s_{i-1}^{l}+B$9:        $d_{s_{i}^{l+1}}$=${∥a\left(s_{i-1}^{l+1}+1:s_{i}^{l+1}\right)-\omega \left(\mu ,s_{i}^{l+1}-s_{i-1}^{l+1}\right)∥}^{2}$10:        calculate all possible $d_{s_{i}^{l+1}}$11:        where $s_{i}^{l}-B\leq s_{i}^{l+1}\leq s_{i}^{l}+B$12:        $s_{i-1}^{*},s_{i}^{*}\leftarrow \arg\min\left\{D_{s_{i-1}^{l+1}}+d_{s_{i}^{l+1}}\right\}$13:        $D_{s_{i}^{l+1}}\leftarrow D_{s_{i-1}^{*}}+d_{s_{i}^{*}}$14:        $S_{i}^{l+1}=S_{i-1}^{*}\cup \left\{s_{i}^{*}\right\}$15:    **end for**16:**end while**17:**return** $S^{l+1}$


The time intervals between successive heartbeats are extracted from the last output S when the algorithm converges. It is necessary to define the convergence condition properly to decide whether to exit after each iteration is completed. It is noted that after several iterations, most of the cut-off points will become stable. For a certain cut-off point, its position will remain same. The convergence condition of the algorithm is defined as follows according to this characteristic.

(15) $$\frac{\#\left\{\left|S_{j}^{l+1}-S_{j}^{l}\right|\leq \alpha_{1}\right\}}{\#\left\{S^{l+1}\right\}}\geq \alpha_{2}$$

where $S_{j}^{l+1}$ represents the *j*-th element of the set $S^{l+1}$, $S_{j}^{l}$ is the corresponding element of the set $S^{l}$, and #{S} represents the number of elements in S. If the difference between the two is less than a predefined threshold $\alpha_{1}$, the cut-off point is considered stable. When the proportion of stable cut-off points in all cut-off points reaches a threshold $\alpha_{2}$, the segmentation converges. In our study, considering that the time interval between two adjacent sampling points is 4 ms, the value of $\alpha_{1}$ can be set to 5 ms to detect the stable points. The value of $\alpha_{2}$ is set to 0.80.

**Figure 6:**
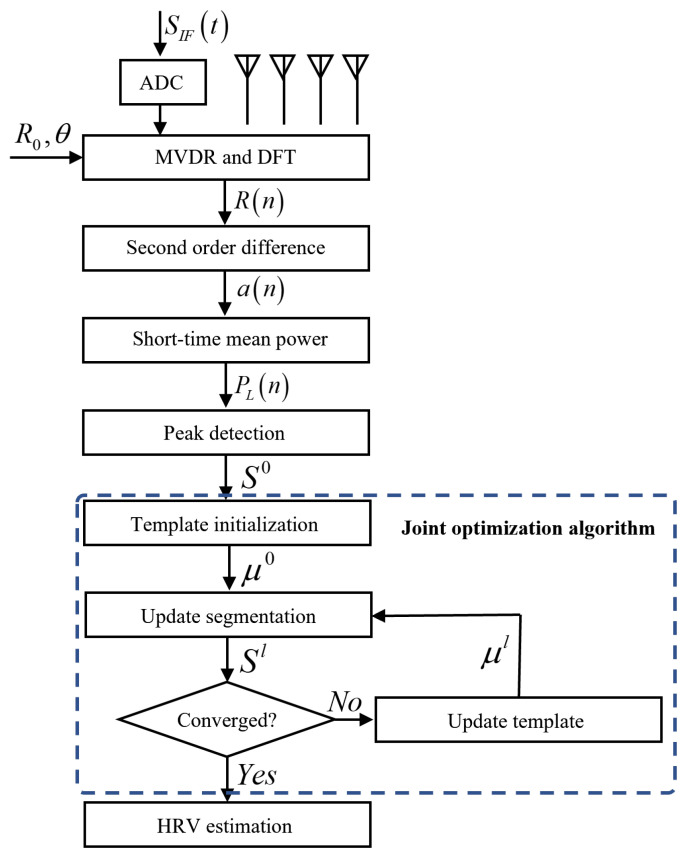
The flowchart for HRV estimation based on radar echoes.

The overall algorithm flowchart for HRV estimation based on radar echoes is shown in Figure 6. The chest wall motion signal R(n) is extracted through MVDR and range FFT based on $R_{0}$ and θ. The acceleration a(n) is calculated to enhance the heartbeat signal and smoothed by short-time average power. The positions of cut-off points are first estimated by the smoothed waveform and then adjusted by a joint optimization algorithm. Finally, the time intervals of successive heartbeats are obtained, and the HRV analysis is performed.

## 3. Experiments

A microwave radar evaluation module (EVM) produced by Texas Instruments (TI), IWR1843, was used to conduct the experiments. It has 3 transmitting antennas (Tx) and 4 receiving antennas (Rx). Each transmitting antenna can transmit 77–81 GHz RF signals. In the experiments, the start frequency of a chirp was set to 77 GHz, and the slope of frequency over time was 70 MHz/μs. Each chirp lasted 50 μs, during which 128 points were sampled. The fast time axis sampling frequency was set to 4 MHz, while the slow time axis sampling frequency was 100 Hz. Tx1, Tx2, and Tx3 transmitted a chirp as described above, in turn, during each frame. The patch antenna array had a peak gain of 10 dB, and the 3 dB beamwidth was approximately $\pm 28^{\circ }$. The mean power density was approximately 1 W/$m^{2}$, which complies with the Exposure Limits in Council Recommendation 1999/519/EC. A DCA1000 EVM was used for radar data sampling, which is a capture card for interfacing with TI’s 77 GHz IWR1843 EVM and enables users to stream the ADC data over Ethernet. The signal interface between the capture card and the IWR1843 EVM uses a 60-pin high-density connector.

**Figure 7:**
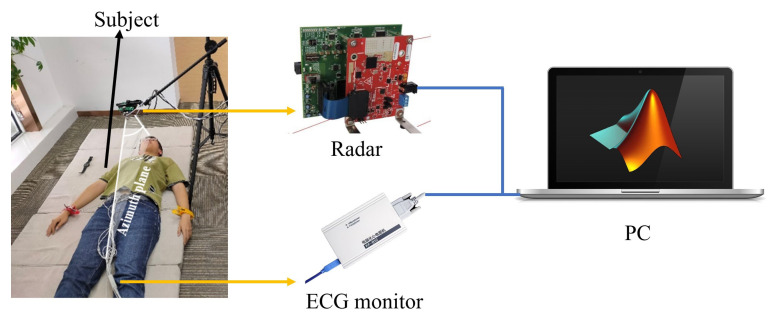
Indoor experimental setup for HRV estimation.

Figure 7 shows the experimental setup for indoor lab testing and evaluation. The FMCW radar sensor was fixed 60 cm above the mattress by using a camera support. To measure the ground truth of IBIs, ECG monitoring was used. A total of 10 electrodes, including 4 attached to the extremities and 6 attached to the chest, corresponding to Wilson leads, were pasted on the body of the monitored subject.

The experiment recruited 10 human subjects (8 male and 2 female; age: 28 ± 6 years; height: 175 ± 10 cm; weight: 58 ± 10 kg) with no respiratory or cardiovascular system diseases. The study was approved by the Medical Ethics Committee of Zhejiang Hospital (Approval Letter No.: AF/SC-06/04.2). The subjects were asked to lie down on the mattress and keep their torso still but breathe normally. The distance and azimuth of subjects’ hearts were recorded at the beginning of the experiments, which were used to find the corresponding range bin and perform beamforming. When the subject was ready, the radar sensor was activated to start sampling first, followed by the ECG monitor. Although the sampled data of the two are not completely synchronized in time, data alignment can be easily achieved by observing the time intervals of each heartbeat and adjusting over a small range (e.g., 5 s). During the experiment, several sets of results with a length of 15 s were acquired for each subject. Four sets of data for each subject were retained after removing the poor-quality fragments. The data were exported to and processed by MATLAB. Note that the sampling frequency was changed to 250 Hz by interpolation before using the proposed algorithm to improve measurement accuracy.

## 4. Results and Discussion

### Results

**Figure 8:**
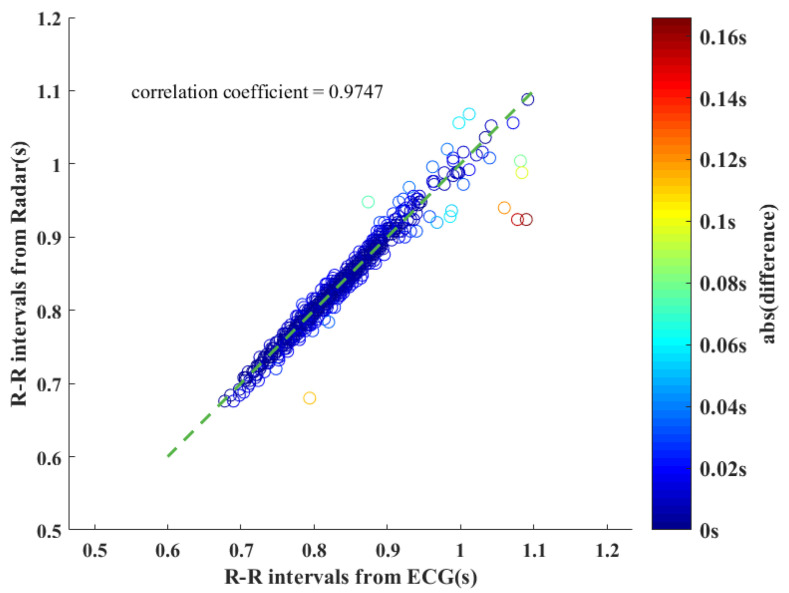
The scatterplot of IBI measurement results from ECG and radar.

From 10 subjects, a total of 40 sets of data containing 694 heartbeats were obtained. Figure 8 shows all IBI measurement results. The scatter is plotted for each heartbeat, using the IBIs extracted from ECG as the horizontal axis and IBIs extracted from radar by the proposed algorithm as the vertical axis. The correlation coefficient between them is 0.9747. It can be seen that most of the points fall near the $45^{\circ }$ oblique line, indicating that the proposed algorithm has high measurement accuracy. The measurement error err of each IBI, defined in (16), was calculated to quantify the measurement accuracy.

(16) $$err\left(n\right)={IBI}_{radar}\left(n\right)-IBI_{ECG}\left(n\right)$$

where $IBI_{radar}\left(n\right)$ and $IBI_{ECG}\left(n\right)$ are the interbeat interval of the *n*-th heartbeat extracted from radar and ECG, respectively.

**Figure 9:**
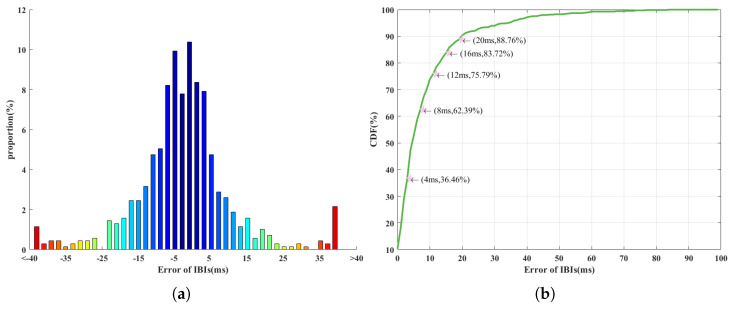
(**a**) The distribution and (**b**) the CDF of measurement errors.

Figure 9a describes the distribution of the measurement error. Most of the measurements have an error of ±2 ms, and the number of measurement points decreases for a large absolute value of error. The whole distribution is almost symmetric around zero error, indicating that the source of the measurement error is more likely a random error. Figure 9b shows the cumulative distribution function (CDF) of measurement results using the absolute value of the measurement error. Note that 36.46% of all the measurement points have an error within ±4 ms. When the allowed error range is enlarged to ±8 ms, over 60% (62.39%) of all points will meet the requirements. More than 80% of all measurement points are within 16 ms of the true value.

The root mean square error (RMSE) of IBIs is calculated by using (17).

(17) $$\mathrm{RMSE}=\sqrt{\frac{\sum_{n=1}^{N}{\left({\mathrm{IBI}}_{radar}\left(n\right)-{\mathrm{IBI}}_{\mathrm{ECG}}\left(n\right)\right)}^{2}}{N}}$$

where *N* is the total number of heartbeats recorded. The RMSE of the proposed algorithm is 14.9 ms for IBI measurements.

The HRV features can be further obtained from IBIs. The three most widely used time-domain metrics are used to evaluate HRV. MEAN is the average value of IBIs, which can be calculated by using (18).

(18) $$\mathrm{MEAN}=\frac{\sum_{n=1}^{N}\mathrm{IBI}\left(n\right)}{N}$$

The standard deviation of normal to normal (SDNN) measures the standard deviation of all IBIs and can be calculated by using (19).

(19) $$\mathrm{SDNN}=\sqrt{\frac{\sum_{n=1}^{N}{\left(\mathrm{IBI}\left(n\right)-\mathrm{MEAN}\right)}^{2}}{N}}$$

The root square of successive differences (RMSSD) measures the successive IBI changes and can be calculated as in (20).

(20) $$\mathrm{RMSSD}=\sqrt{\frac{\sum_{n=2}^{N}{\left(\mathrm{IBI}\left(n\right)-\mathrm{IBI}\left(n-1\right)\right)}^{2}}{N-1}}$$

**Figure 10:**
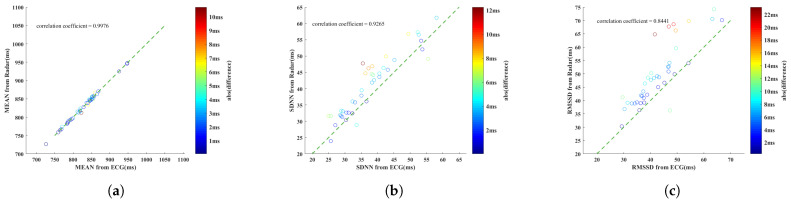
The scatterplot of (**a**) MEAN, (**b**) SDNN, and (**c**) RMSSD estimates from ECG and radar.

The above three metrics were calculated for each set of sampling data, and the scatter of the results is drawn in the same form as Figure 8. Figure 10a shows the estimation results of MEAN. The correlation coefficient is 0.9976, indicating that the two have a strong correlation. The RMSE of MEAN in all 40 sets of data is 3.24 ms. Figure 10b,c show the estimation results of SDNN and RMSSD, respectively. Most of the points are above the oblique line, indicating that the corresponding HRV metrics calculated based on the RF signal are relatively large, which is more obvious for RMSSD than for SDNN. The RMSE in all 40 sets of data is 4.91 ms for SDNN and 9.10 ms for RMSSD.

The HRV estimation results of 10 experimental subjects are listed in Table 1. One set of sampling data was selected from four sets for each subject. The estimated results of MEAN obtained by the proposed algorithm are very close to the true values, with the estimation errors of all 10 subjects being less than 3 ms. For SDNN, 9 of 10 experimental subjects’ estimation errors are within 5 ms, and the maximum error is 5.4 ms. For RMSSD, the estimation errors of 9 subjects are within 10 ms, and the maximum estimation error is 10.9 ms.

## 5. Discussion

As an important method for the noncontact detection of human physiological parameters, biological radar collects the vibrations outside the human heart from RF signals, analyzes the phase changes, extracts heartbeats, and estimates the corresponding parameters through signal processing algorithms. In this article, a set of algorithms is proposed for HRV monitoring via FMCW radar. The hardware platform we use actually has a sampling frequency of 100 Hz, which means that the interval between two adjacent sampling points in the heartbeat waveform is 10 ms. The experimental results show that the measurement errors of the proposed algorithm for the HRV indexes are less than 10 ms, so we believe that the accuracy is satisfactory.

The measurement of the time intervals of heartbeats based on RF methods depends largely on the quality of echo signals and a high signal-to-noise ratio (SNR) of the heartbeat signal. Radar equipment with better performance and measurement constraints, such as the position relative to the radar and the body postures of the subject, are usually required to obtain higher-quality signals. Locating the radar close to the heart usually leads to higher signal quality. In some systems, such as the PhysioChair [40], the stress assessment method [41], and the method for monitoring a driver’s heartbeat information [42], the radar is installed in the position of the seat back directly opposite to the chest, and obvious heartbeat signals can be seen in the chest movement waveform demodulated from radar echoes. A simple processing method like a bandpass filter is sufficient to extract heartbeat signals with high quality. However, it is difficult to objectively evaluate the effects of various HRV monitoring methods based on millimeter-wave radar with different RF equipment and in different monitoring conditions. Therefore, we selected several relevant studies that used the same or very similar RF devices as we used and compare these studies in Table 2, where, ’Distance’ represents the distance between the antenna and the subject, and ’HR ACC’ represents the accuracy of heart rate measurement.

Compared with the existing common HRV extraction algorithms, such as the heartbeat extraction algorithm based on decomposition [46], which has an RMSE of MEAN, SDNN, and RMSSD of 4.65 ms, 10.31 ms, and 9.18 ms, respectively, the proposed algorithm achieves higher accuracy, with the RMSE being 3.24 ms, 4.91 ms, and 9.10 ms. Although the stochastic resonance algorithm [30] can estimate SDNN with a relative error of less than 6.53%, it is not ideal for some HRV indices (such as pNN50), as it cannot accurately measure the time duration of each heartbeat. The algorithm based on Gaussian pulse train modeling and frequency–time phase regression (FTPR) [34] has a similar problem.

**Figure 11:**
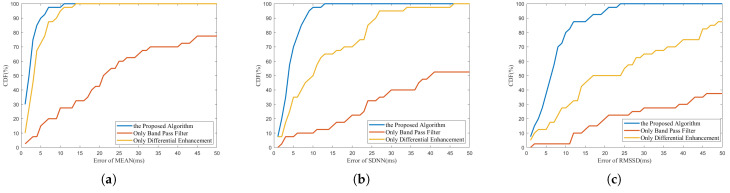
The error CDF curve of (**a**) MEAN, (**b**) SDNN and (**c**) RMSSD index obtained by different algorithms.

A bandpass filter (BPF) is a very common method to extract heartbeat signals from radar signals. It is also used in HRV monitoring based on millimeter-wave radar [23,34]. However, the extraction effect is significantly affected by the quality of the original data. To compare the proposed algorithm with the algorithm based on the bandpass filter, a 0.8–1.5 Hz IIR bandpass filter was designed to process the same 40 sets of data. The measurement errors between MEAN, SDNN, and RMSSD obtained from each group of data and the gold standard were calculated. The CDF of errors is shown in Figure 11a–c. It can be seen that the accuracy of the HRV index calculated by the proposed algorithm is significantly higher than that calculated by IBIs extracted by the bandpass filter. For the MEAN index, the measurement error of 90% of the experimental data is less than 5 ms, and the maximum error is less than 15 ms. However, only 15% of the experimental data with bandpass filters have measurement errors of less than 5 ms, and even more than 20% of the experimental data have measurement error greater than 50 ms. For the SDNN index and the RMSSD index, the proposed algorithm can also achieve measurement errors of less than 13 ms and 24 ms, but for the bandpass filter, nearly half (47.5%) of the experimental data have SDNN index measurement errors exceeding 50 ms. Only 37.5% of the experimental data have RMSSD index measurement errors within 50 ms. It can be concluded that the HRV index calculated based on the heartbeat signal extracted by a simple bandpass filter has a large error, and its accuracy cannot meet the monitoring needs of the HRV index considering that the normal values of SDNN and RMSSD of normal people are usually tens of milliseconds.

Calculating the acceleration of the chest wall motion signal to enhance the heartbeat signal is a key part of the proposed algorithm. A similar method was also adopted in [38], showing a good ability to suppress the harmonics of breath and the potential for HRV monitoring. However, the HRV of only one set of data was analyzed in their experiment, and the relative error was more than 30% for RMSSD. It should be pointed out that IBIs with high accuracy cannot be extracted only by the smoothed acceleration signal. That is, the optimization of segmentation to adjust the time interval of each heartbeat in the proposed algorithm is also necessary. To illustrate this point, the HRV index calculated by IBIs extracted only by differential strengthening and smoothing is also shown in Figure 11a–c. It can be seen that for the MEAN index, the difference between differential enhancement and the proposed algorithm is not obvious, because the position adjustment of segmentation points between each heartbeat does not affect the total duration of all heartbeats. Both methods can achieve measurement errors of less than 15 ms. However, for SDNN and RMSSD, which are more sensitive to the measurement error of each IBI, the proposed algorithm is significantly better than that without the optimization of segmentation. For SDNN, the maximum measurement error obtained by only differential enhancement exceeds 45 ms, while for RMSSD, more than 10% of the results obtained by only differential enhancement have measurement errors exceeding 50 ms. The use of only differential enhancement cannot meet the monitoring accuracy requirements of HRV indexes either, especially for SDNN and RMSSD indexes. Table 3 quantitatively shows the comparison between the proposed algorithm and the above two algorithms in terms of accuracy, where BPF represents the algorithm based on the bandpass filter, and DE represents the algorithm with only differential strengthening without the optimization of segmentation.

The algorithm in [39] achieved a good measurement accuracy for IBI measurement with an average error of 3.2 ms. However, the amount of computation required by this algorithm is very large, making it difficult to monitor in real time. It took several minutes to run the corresponding script on an ordinary PC to process a set of 15 s sampling data. The computational complexity problem also exists in the algorithm in [35]. It achieved an average error of 12.2 ms for IBI measurement and successfully realized myocardial infarction detection based on HRV. However, this algorithm contains a lot of mode decomposition and optimal segmentation. The proposed algorithm achieves a comparable measurement error with much less computation. The average measurement error of the proposed algorithm is 9.3 ms, and it only takes a few seconds to complete a set of 15 s sampling data by running the script of the proposed algorithm under the same conditions. The proposed algorithm achieves a better balance between accuracy and computational complexity, which shows the potential of real-time monitoring. This study also reveals the potential of the proposed algorithm for cardiovascular system diagnosis. We noticed that most of these products claim that they can measure the heart rate with less than 2% error. Table 2 shows that the accuracy of our algorithm for heart rate measurement is higher than 98%, indicating that the proposed algorithm has the potential for clinical application in terms of accuracy. It should be pointed out that the above accuracy was obtained on the basis of data measured in a laboratory environment, which is relatively simple, and the number of experimental subjects was relatively small. A larger range of experimental verification is required before clinical application.

**Figure 12:**
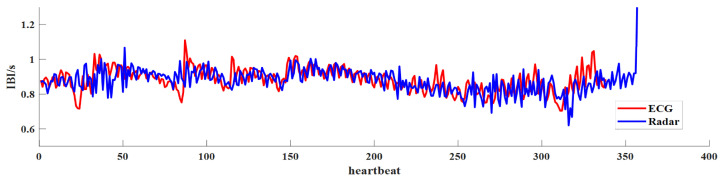
The extracted IBIs from radar and ECG when there are two individuals in the FOV.

The proposed method also has some limitations. Firstly, its anti-interference capability is still limited in some cases, such as when monitoring multiple targets at the same time. Figure 12 shows the IBIs of one target when there are two individuals in the field of view (FOV) of the radar. It can be seen that the proposed algorithm does not extract the IBIs very well in this case. This may be due to the superimposition of chest wall motion signals from two individuals. Although MVDR beam-forming technology was adopted in our algorithm to suppress interference from other directions, because the number of antennas in our hardware equipment is limited, the echo signal from another individual is not completely suppressed when extracting the heartbeat information of one of the monitored targets. In addition, it was found in the experiments that when subjects cough or scratch, large interference will be introduced into the acceleration signal, leading to a significant deviation of the IBI estimation results from the real value. We also noticed that for a long period of data (e.g., 5 min), the measurement effect of this algorithm for IBIs is not ideal, because the acceleration waveform of a single heartbeat will also change over time. Therefore, it is difficult to find a unified template signal to segment the long period of acceleration.

## 6. Conclusions

This paper proposes an algorithm for HRV monitoring. Firstly, it extracts the echo signal of the heart through the distance and azimuth angle relative to the antenna array parameters, which are measured before the experiments. The acceleration of the chest wall motion signal is calculated to suppress respiration and strengthen the heartbeat. Then, the acceleration is smoothed by short-time average power, from which the cut-off points are estimated. Finally, IBIs are extracted by a joint optimization algorithm, and HRV features are estimated. Experimental results show that this algorithm can achieve good measurement accuracy in a laboratory environment. However the measurement performance degrades in a complex real scenario or a long-time monitoring scenario. In an environment that is not suitable for ECG and PPG measurement, the measurement method based on RF technology can provide a new approach for the measurement and analysis of HRV. In the future, we plan to apply the proposed algorithm to sleep monitoring. The trunk of the subject is relatively still during sleep time, so the HRV indicators can be estimated more accurately. We wish to help detect some diseases, such as OSA, by monitoring the HRV indicators of subjects during sleep.

## Figures and Tables

**Table 1:** HRV estimation results in terms of MEAN, SDNN, and RMSSD for 10 subjects.

Subject ID	MEAN (ms)			SDNN (ms)			RMSSD (ms)		
	Radar	ECG	Err	Radar	ECG	Err	Radar	ECG	Err
1	945.6	947.2	1.5 (0.16%)	54.7	52.3	2.4 (4.65%)	65.4	63.2	2.2 (3.46%)
2	809.0	809.5	0.5 (0.06%)	35.6	31.7	3.9 (12.3%)	52.5	44.3	8.3 (18.7%)
3	758.7	759.4	0.7 (0.09%)	44.7	40.6	4.1 (10.2%)	48.7	41.3	7.3 (17.8%)
4	812.6	815.3	2.6 (0.33%)	43.7	38.2	5.4 (14.2%)	48.5	40.3	8.1 (20.2%)
5	786.0	786.4	0.4 (0.05%)	33.9	32.3	1.6 (5.00%)	38.0	35.9	2.1 (5.96%)
6	864.0	865.1	1.1 (0.12%)	44.3	42.6	1.7 (3.94%)	52.1	48.7	3.5 (7.11%)
7	853.9	854.7	0.8 (0.09%)	33.9	31.2	2.7 (8.81%)	41.2	36.5	4.7 (12.8%)
8	850.3	851.3	0.9 (0.11%)	44.5	39.6	4.9 (12.3%)	57.5	46.6	10.9 (23.5%)
9	846.7	847.1	0.3 (0.04%)	37.7	33.6	4.1 (12.2%)	56.8	47.3	9.6 (20.2%)
10	850.5	850.8	0.3 (0.04%)	44.1	39.5	4.6 (11.7%)	63.9	55.0	8.8 (16.1%)
Average		0.91 (0.11%)			3.5 (9.53%)			6.6 (14.6%)	

**Table 2:** Comparison of the heartbeat signal monitoring results with the results of other works.

Ref.	RF Equipment	Distance (m)	HR ACC. (%)	HRV Monitoring	IBI RMSE (ms)
[43]	AWR1642 (TI)	0.5	⩾90%	No	-
[44]	IWR1443 (TI)	0.28–0.7	97%	No	-
[45]	IWR1843 (TI)	0.5–2.0	98%	No	-
[46]	IWR1843 (TI)	0.5	Not Mentioned	Yes	26.06
Ours	IWR1843 (TI)	0.5	⩾98%	Yes	14.9

**Table 3:** Comparison of three algorithms in measurement accuracy.

The Proportion of Measurements with a Measurement Error of	MEAN			SDNN			RMSSD		
	BPF	DE	Ours	BPF	DE	Ours	BPF	DE	Ours
⩽5 ms	15.0%	72.5%	90.0%	7.5%	35.0%	70.0%	2.5%	12.5%	37.5%
⩽10 ms	27.5%	95.0%	97.5%	10.0%	50.0%	97.5%	2.5%	27.5%	80.0%
⩽15 ms	32.5%	100%	100%	15.0%	65.0%	100%	12.5%	45.0%	87.5%
⩽20 ms	42.5%	-	-	22.5%	70.0%	-	20.0%	50.0%	95.0%
⩽30 ms	65.0%	-	-	40.0%	95.0%	-	27.5%	65.0%	100%
⩽40 ms	70.0%	-	-	50.0%	97.5%	-	30.0%	75.0%	-
⩽50 ms	77.5%	-	-	52.5%	100%	-	37.5%	87.5%	-
RMSE (ms)	46.30	4.92	3.24	88.21	16.36	4.91	117.70	31.54	9.10

## Data Availability

Data will be made available on request.

## Abbreviations

The following abbreviations are used in this manuscript:

HRV	Heart Rate Variability
FMCW	Frequency-modulated continuous wave
IBI	Interbeat interval
RMSE	Root mean square error
SDNN	Standard deviation of normal to normal
RMSSD	Root mean square of successive differences
ANS	Autonomic nervous system
PNS	Parasympathetic nervous system
SNS	Sympathetic nervous system
ECG	Electrocardiography
PPG	photoplethysmography
OSA	obstructive sleep apnea
SIDS	Sudden infant death syndrome
CW	Continuous wave
EMD	Empirical mode decomposition
IMF	Intrinsic mode function
DE	Differential enhancement
HR	Heartbeat rate
MIMO	Multiple input multiple output
IF	Intermediate frequency
ADC	Analog-to-digital converter
FFT	Fast Fourier transform
MVDR	Minimum variance distortionless response
EVM	Evaluation module
Tx	Transmitting antennas
Rx	receiving antennas
CDF	Cumulative distribution function
FTPR	Frequency time phase regression
BPF	Bandpass filter
FOV	Field of view
